# As-needed endotracheal suctioning protocol vs a routine endotracheal suctioning in Pediatric Intensive Care Unit: A randomized controlled trial.

**DOI:** 10.25100/cm.v49i2.2273

**Published:** 2018-06-30

**Authors:** Gloria Lucía Lema-Zuluaga, Mauricio Fernandez-Laverde, Ana Marverin Correa-Varela, John J. Zuleta-Tobón

**Affiliations:** 1 Epidemiology Academic Group (GRAEPIC), Universidad de Antioquia, Medellin, Colombia.; 2 Research Unit, Hospital Pablo Tobón Uribe, Medellín, Colombia; 3 Hospital Pablo Tobón Uribe, Medellín, Colombia; 4 Universidad CES, Medellin, Colombia.

**Keywords:** Suction, Intubation, endotraqueal, adverse effects, pediatric, randomized controlled trial, arrhythmias, cardiac, hypoxia, aspiración endotraqueal, eventos adversos, población pediátrica, ensayo clínico controlado, arritmias cardiacas, hipoxia

## Abstract

**Objective:**

To compare two endotracheal suctioning protocols according to morbidity, days of mechanical ventilation, length of stay in the Pediatric Intensive Care Unit (PICU), incidence of Ventilator-Associated Pneumonia (VAP) and mortality.

**Methods:**

A Pragmatic randomized controlled trial performed at University Hospital Pablo Tobón Uribe, Medellin-Colombia. Forty-five children underwent an as-needed endotracheal suctioning protocol and forty five underwent a routine endotracheal suctioning protocol. Composite primary end point was the presence of hypoxemia, arrhythmias, accidental extubation and heart arrest. A logistic function trough generalized estimating equations (GEE) were used to calculate the Relative Risk for the main outcome.

**Results:**

Characteristics of patients were similar between groups. The composite primary end point was found in 22 (47%) of intervention group and 25 (55%) children of control group (RR= 0.84; 95% CI: 0.56-1.25), as well in 35 (5.8%) of 606 endotracheal suctioning performed to intervention group and 48 (7.4%) of 649 performed to control group (OR= 0.80; 95% CI: 0.5-1.3).

**Conclusions:**

There were no differences between an as-needed and a routine endotracheal suctioning protocol.

**Trial registration:**

ClinicalTrials.gov identifier NCT01069185

## Resumen

**Objetivo:**

Comparar dos protocolos de aspiración endotraqueal con respecto a morbilidad, días de ventilación mecánica, duración de la estancia en la Unidad de Cuidados Intensivos Pediátrica (UCIP), incidencia de neumonía asociada al ventilador (NAV) y mortalidad.

**Métodos:**

Ensayo clínico controlado pragmático realizado en el Hospital Universitario Pablo Tobón Uribe, Medellín-Colombia. Cuarenta y cinco niños tratados con un protocolo de aspiración endotraqueal a necesidad y 45 con un protocolo de aspiración endotraqueal rutinaria. El desenlace primario compuesto fue la presencia de hipoxemia, arritmias, extubación accidental y paro cardiorrespiratorio. Se utilizó un modelo de ecuaciones estimables generalizadas (GEE) para calcular el riesgo relativo para el desenlace principal.

**Resultados:**

Las características basales fueron similares entre los grupos. El desenlace primario compuesto estuvo presente en 22 (47%) niños del grupo de intervención y 25 (55%) niños del grupo control (RR= 0.84; IC 95%: 0.56-1.25), así como en 35 (5.8%) de las 606 aspiraciones endotraqueales realizadas al grupo de intervención y en 48 (7.4%) de las 649 realizadas al grupo control, (OR= 0.80; IC 95%: 0.5-1.3).

**Conclusiones:**

No existen diferencias entre un protocolo de aspiración endotraqueal y uno a necesidad.

## Introduction

The percentage of intubation and mechanical ventilation in the pediatric intensive care unit (PICU) varies between 17% and 64% with an median duration of 7 days^1^. The presence of the endotracheal tube (ETT) and the inadequate humidification of the inspired air may cause irritation of the airways with the consequent increase in secretion production. The ineffective mucociliary function because of sedation and compromise for glottic closure due to the presence of the ETT, affect the cough mechanism causing retention of secretions, airway obstruction, increase airway resistance, decrease of ventilation and air flow and finally increase in the respiratory efforts. To prevent the previous manifestations, it is necessary to remove the secretions accumulated in the ETT, the trachea and the low respiratory airways using endotracheal (ET) suctioning ^2^. Even though the technique is relatively simple, several studies have described adverse clinical events associated to this procedure, from which the following stand out: hypoxemia, with a decrease of oxygen saturation of up to 30%, presence of arrhythmias, cyanosis, changes in the blood arterial pressure, risks of introducing pathogens into the respiratory tract, accidental extubation and in some cases, although not frequent, death ^3^.

In the literature there are recommendations regarding the pressure of the suction, the suction systems, the duration of the procedure, the depth of the insertion of the catheter and its size, but there is controversy concerning the frequency to apply the ET suctioning, such as its impact on the duration of the respiratory support, the length of stay in the PICU, the incidence of infections associated to the health care and the accidental extubations^2^. The latest guidelines of the “American Association of Respiratory Care” (AARC), recommend with a high degree but with low quality of evidence, the use of the ET suctioning only in the presence of secretions^4^.This recommendation was based on three clinical trials, one conducted in adults and the other two in children with very-low-birth-weight^5^ ^-^ ^7^, all implemented more than 12 years ago.

Taking into account the multiple risks that represent the ET suctioning for the pediatric population, which increases with its frequency, the fact of decreasing the number of suctions could be reflected in a decrease of accidental extubations and its consequences, such as the negative inherent effects from the suctions.

In this clinical trial, both protocols of ET suctioning were compared, to identify if there are differences in the frequency of the morbidity associated to this, days of mechanical ventilation, the length of stay in the PICU, the incidence of Ventilator-Associated Pneumonia (VAP) and mortality in the PICU.

## Materials and Methods

### Study design and participants

This pragmatic, parallel, unblinded, randomized controlled trial considered for random allocation, all children over a month up to 14 years plus 364 days, hospitalized in the PICU who required endo-tracheal intubation and mechanical ventilation, whose parents accepted to sign the waived informed consent. Excluded from the study were those patients with high-frequency oscillatory ventilation, due to the depressurization risk because of suctioning and the difficulty of visualizing the pattern of presence of secretions in children submitted to this type of ventilation.

This study was conducted over a six-month period at University Hospital Pablo Tobón Uribe, located in Medellín-Colombia, which has 313 beds, 10 in pediatric ICU with 72 discharges per year. The study protocol was reviewed and approved by Ethics Committee of the Hospital. 

### Random Allocation

Group allocation to either as-needed or routine ET suctioning protocol, was obtained using The RAND software by random sequence numbers distributed over permutated blocks size of 4,6 and 8 generated by the head investigator who was not involved in patient care. The patients were randomized immediately after intubation. The allocation concealment of the sequence was applied by sequentially numbered, sealed, opaque envelopes kept by Hospital’s Research Unit.

### Interventions

The guides of aseptic technique for ET suctioning practice were considered. After obtaining the informed consent from parents, the practice of the ET suctioning was applied in both groups using a Trach Care^TM^ catheter which was introduced in the ETT avoiding contact with the airway and applying negative pressure, between 20 and 40 mmHG, according to patient’s age, for a maximum period of 10 seconds. This technique was repeated until the airway remained patent which was assessed by the evidence of non-secretions in the aspirator as well as in the flow pattern. The size of the catheter was protocolized. As a rule, we assigned the catheter with double the number of the tube the child has: for a number 3 tubes we use a number 6 catheter, for a number 4 tubes we use a number 8 catheter ^2^.

In the intervention group, the practice of ET suctioning was applied as-needed according to the presence of one or several of the following criteria displayed on the ventilator monitor or bydirect observation:

- Oxygen saturation less than 90% (arterial desaturation) not secondary to movement or loss of the pulse wave, for duration of at least one minute and with previous normal saturation.
- Tidal volume less than or equal to 5 mL/kg of what the patient mobilized before the ES and whose decrease was not secondary to a previous manipulation. For each patient the pediatrician on duty calculated the absolute ideal tidal volume in mL/kg and the minimum allowed tidal volume, which was programmed in the ventilator alarm. A fall in the tidal volume was considered having as reference the absolute ideal tidal volume that the patient had. This value was taken from the evolution notes of the respiratory therapist and the pediatrician. Pressure parameters like PEEP and pressure control were not considered.
- Curve of flow/time that showed changes of graphics or the presence of “saw-toothed pattern” which indicated the presence of secretions in ETT. The presence of water in the ventilator tubing was ruled out.
- Asynchrony of the respiratory pattern with the ventilator: irregular and uncoordinated respiratory pattern with the ventilatory mode.
- Audible and visible secretions.

In the control group, the practice of ET suctioning was performed as a routine form every two hours. In this group the frequency of suction could be modified if the patient presented whichever criteria defined for the intervention group, remaining in the assigned treatment arm. There was no additional sedation applied during the practice of ET suctioning. It should be noted that an active moistening servo controlled by humidifier Fisher and Pyker MR 290 was used.

Due to the inability to blind the intervention, the frequency of the following co-interventions that were part of basic nursing care, which could be modified by the group knowledge and at the same time could affect the expected results, were quantified: changes of position, bath in bed, fixation of the ETT and sedation. A staff different from the research team performed this evaluation.

### Outcomes

A primary end point of morbidity was established, which was composed by four components defined as follows:

- Hypoxemia as the drop in saturation of 10% of the previous value to the ET suctioning, presented during or immediately ending the suction.
- Accidental extubation events Cardio respiratory arrest.
- Arrhythmias as the alteration of heart rhythm-based, presented during or immediately ending the suction and considered as: bradycardia if heart rate was below the 5^th^ percentile for patient’s age or tachycardia if heart rate was greater than 90^th^ percentile for patient’s age or if there was an increase of 10% for those patients that had tachycardia before the suction.
- Extrasystoles like the presence of premature beats compared to the normal rate, identified by the nursing assistant, verified by the pediatrician in monitors. The data for the registration of these adverse events were collected from the central monitor CNS Nihon Kohden V 01-55 9701J with the help of the Vorax 5001 software Spanish version.

Secondary outcomes were defined as follows:

- Ventilator-Associated-Pneumonia (VAP) whose definition was based on the criteria of the “Center Disease Control” (CDC) in Atlanta and its diagnosis was checked by the Infections Prevention Committee.
- Days of mechanical ventilation and stay in the PICU accounted every day at 7 hours and 30 minutes. This measurement was the duty of the Infections Prevention Committee from Monday to Friday. During weekends and holidays, the person in charge of the measurements was the nurse on duty. For the calculation, it was taken into account the date, time entry and discharge in the PICU in the registration of the electronic health record.
- Measurements of the cardiopulmonary parameters were done by the nursing assistant, the nurse, the respiratory therapist or the pediatrician and were recorded in a standardized registration sheet. A different physician from the treating group assigned the diagnosis according to analyses done on such data. There were considered additional variables such as: The age in months, the gender, the weight in kilograms, the diagnosis of the PICU admission recorded in the electronic health record by the pediatrician and the PRISM (Probability Risk Infant Score Mortality) calculated for the first 24 hours of admission to the PICU.

Outcomes included were the events occurred during the PICU stay only.

### Sample size

Calculation was based on findings from the literature. We assumed a proportion of events in the control group of 45% and an expected decline of 25% in the intervention group ^8^ ^,^ ^9^. The alpha error was set a significant level of 0.05 and the beta error at 0.20. Taking into account that it was about repeated measurements, sample size was done with an intracluster coefficient correlation (ICC) of 0.1. Hoping to have a minimum of 20 suctions for each patient included in the study, the minimum number of patients per group required to fulfill these criteria was 45. The program used for the calculation was Sampsize version 1.0.2.

### Statistical analysis

The randomly assigned units were the patients and the analysis units were the suctions. For those demographic and clinical characteristics of the population represented in dichotomous variables, absolute and relative frequencies were calculated. For continuous variables, the normal distribution of the data was assessed by measures of central tendency, measures of distribution, graphics and the Shapiro-Wilk test. Due to the skewed distribution of the data, medians were used with its respective interquartile range (IQR). For bivariate analysis of categorical variables, the Chi square statistic was used. In those cases were the marginal expected values were lower than 5, continuity correction was applied. Continuous variables were compared with Mann-Whitney test. For both types of variables, a *p* value less than 0.05 was considered statistically significant. To estimate the relative risk of the primary composite end point was considered the fact that responses were correlated. It was necessary to model the data using techniques for dichotomous outcomes by generalized estimating equations (GEE). Within the model, the number that identified each patient in the data base represented the variability between-subject and the measurements ET suctioning represented the variability within-subject. The type of model chosen was the binary logistic response. The dependent variable was the primary composite end point, the category of reference was the control group and as predictable factors the ET suctioning protocol (routine or as-needed) and the number (episodes) of ET suctioning were used. The choice of “working correlation structure” was assessed and the model was chosen considering the lowest values for standard errors and for the quasi-likelihood criteria of goodness-of-fit statistic ^10^. All analysis were by “intention-to-treat-analysis”, all admitted patients remained in the study, regardless of the final duration of the intubation and the program “Statistical Package for the Social Sciences” (SPSS) ^®^ version 19.0 for windows was used.

## Results

**Figure 1:**
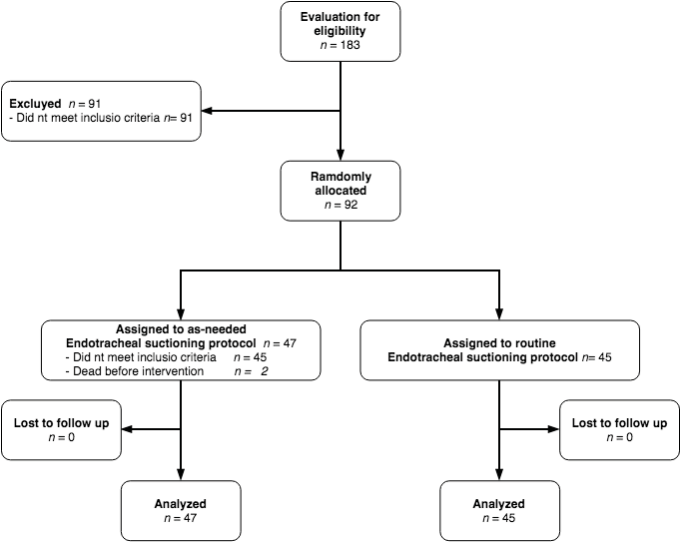
Flow of participants

During the study period, 183 patients were admitted to the PICU. Of these patients, 92 were randomly assigned to receive as-needed or routine ET suctioning protocol. Finally, 45 patients received as-needed ET suctioning and 45 received routine ET suctioning. There was no lost to follow up due to each patient was followed until discharge from the PICU (Fig. 1).

From the recruited population, 52% (48/92) were male patients. Median age was 40.5 months (IQR 8.3 - 107.1). The reasons for admission to the PICU were the infectious diseases in 16% (15), congenital diseases in 12% (11), neurological and surgical diseases in 11% (10), cardio vascular diseases in 9% (8), respiratory diseases in 8% (7) and 34% (31) got admitted for other causes. Of the admitted participants, 23% (21) died. The median of the PRISM score for the randomly assigned population was 22 points (IQR:15-26). Most children (44%) at the admission showed a low risk of death (PRISM >20 points). The median duration of mechanical ventilation and stay in the PICU, was of 2.5 days (IQR:1.0-5.7) and 5.8 days (IQR:2.0-14.6) respectively. Ninety-eight per cent of the participants received sedation, bed bath and position changes according to the nursing care protocol. The protocol of fixation of the ETT was applied to 96% (88) of the participants; as there were four participants who entered with tracheotomy. 

Patient clinical characteristics and demographic data were similar between the two groups (Table 1). The median of ET suctioning for each patient of the intervention and control group was 8 suctions (IQR: 4.0-13 vs 4.0-14).The mean time between each aspiration in as-needed ET suctioning group was 3.8 hours. The primary composite end point was present in 22 (47%) children of the intervention group and 25 children (55%) of the control group (RR= 0.84; 95 % CI= 0.56-1.25). At the end of the follow up, the primary composite end point was presented in 35 (5.8%) of the 606 episodes of ET suctioning in the intervention group and in 48 (7.4%) of the 649 episodes of ET suctioning practiced to the control group (Table 2). After evaluating the different correlation structures, the independent structure was chosen and an OR for the primary composite end point was obtained of 0.8 with an 95% confidence interval of 0.5-1.3 (*p*= 0.31). There was only one case of VAP in the control group without statistical significance between the groups (*p*= 0.98). In the intervention group the median of the length of mechanical ventilation was 2.5 days (1.0-5.0) vs. 2.4 days (0.8-6.4) in the control group, without statistical significance (*p*= 0.93). The length of stay in the PICU was longer for the intervention group with a median of 6.0 days (1.8-12.6) vs. 4.4 days (2.4-22) in the control group (*p*= 0.76). In an exploratory analysis was discovered that 12 (26.7%) participants died in the control group and 9 (19.1%) died in the intervention group (*p*= 0.39) during the intervention period. There was not follow-up after PICU discharge.

**Table 1:** Demographic and clinical characteristics of the patients.

Characteristics	As-needed ET suctioning protocol (intervention) n= 47	Routine ET suctioning protocol (control) n= 45
Men n (%)	26 (55.3)	22 (48.9)
Age months Median (P25-75)	34 (7-104)	56 (14-108)
Weight (kg) Median (P25-75)	15 (7-31)	15 (10-30)
PRISM Median (P25-75)	23 (14-28)	23 (17-27)
Income diagnosis n(%)		
Respiratory diseases	5 (10.6)	2 (4.4)
Neurologic diseases	7 (14.9)	3 (6.7)
Post-surgical procedures	7 (14.9)	3 (6.7)
Congenital malformations	7 (14.9)	4 (8.9)
Cardiovascular diseases	3 (6.4)	5 (11.1)
Infectious diseases	4 (8.5)	11 (24.4)
Others	14 (29.8)	17 (37.8)

**Table 2:** Results

Outcome	As-needed ET suctioning (n= 606)^**§**^	Routine ET suctioning (n= 649)	OR (CI 95%)^**‡**^
Primary composite end point^**†**^	35 (5.8%)	48 (7.4%)	0.8 (0.5-1.3)
Arrhythmias	26 (4.4%)	41 (6.4%)	0.7 (0.4-1.2)
Hypoxemia	10 (1.7%)	7 (1.1%)	1.5 (0.5-4.4)
Accidental extubation events	0	0	
Cardio respiratory arrest	0	0	

§ Number of suctions

‡ OR calculate having account correlation between measures

† Hypoxemia, arrhythmia, accidental extubation and cardio respiratory arrest

## Discussion

This trial suggests that the application of one as-needed ET suctioning protocol compared to one of routine ET suctioning, does not present differences in the frequency of morbidity for the children who were admitted to the PICU. Although indicated to declare equality between two interventions it is necessary to perform a non-inferiority trial, the small difference found between the two groups, with minimal clinical significance, suggests that both interventions are similar with respect to adverse events, consistent with current recommendations of clinical guidelines.

The clinical guidelines of the “American Association Respiratory Care” (AARC) for the practice of the ET suctioning in patients with mechanical ventilation and artificial airway recommend that routine suctioning should be avoided, reinforced by the results of three clinical trials, however, one was conducted on adults ^7^ and two on newborn of very-low-birth-weight with respiratory distress syndrome ^5^ ^,^ ^6^. The frequency of ET suctioning and the outcomes compared in those trials were different than those assessed in our trial. A revision of five old studies, with only one randomized trial, addressing the frequency of suctioning in neonates and infants concluded that there is a paucity of evidence to support suctioning as needed in neonates and infants ^11^. The findings in our research provide additional information for a pediatric population, for whom suctioning frequency was not previously evaluated.

There exist several observational studies in the pediatric population, which have described the incidence of adverse events associated to the practice of the ET suctioning, and have been reviewed recently ^12^. Some authors have described that hypoxemia is the most frequent adverse event during the practice of ET suctioning (20%) ^3^ ^,^ ^13^, this finding differs from what was found in our trial, where the arrhythmias, mainly the bradycardia, was the most frequent complication as has been reported in other studies ^14^ ^,^ ^15^.

The absence of accidental extubation during the practice of the ET suctioning in this trial differs from the others reported , where the practice of procedures like the ET suctioning was described like one of the risk factors for accidental extubation and it has been described that ET manipulation at the time of unplanned extubation in the neonatal ICU ranged from 17% to 30% of all patients who had unplanned extubation ^16^. Some authors have described the increase of days of mechanical ventilation and stay in the PICU as a consequence of the accidental extubation ^17^. Our trial did not show differences in both durations, one of the aspects that could influence this result could be the absence of accidental extubations. The differences in the frequency of the secondary outcomes of our trial with the observational studies possibly are based on methodological issues.

There were no additional efforts or necessity for more personal to practice the “as needed protocol”. Using this protocol, the time between aspirations were doubled. The released time was using for provide other cares that gave more benefits for the patients. However, this outcome was not objectively measured having account it was not the aim of the study. 

One limitation of this study was the low frequency for the primary outcome, lower than estimated from those reported in the literature, which affects the power of the study, however, the difference found between the two groups is very small and was also lower than estimated from observational studies, therefore is considered to be plausible to accept that the failure to detect significant differences in the frequency of endpoints between the two groups was not due to the decline in power of the study. A second limitation was the lack of blinding, which is impossible to avoid considering the type of intervention implemented, but tried to control by assessing the outcome by staff independent of the research team and indirectly evaluating the possibility that this knowledge would induce other differential co-interventions, which did not appear. There was also difficulty to define objectively the components of the primary outcome. 

A recent Cochrane review of frequency of endotracheal suctioning for the prevention of respiratory morbidity in ventilated newborns find that the ideal frequency of ET suctioning has been investigated in few studies, identified only one randomized controlled study from 1991for inclusion in the revision and concluded that there was insufficient evidence to identify the ideal frequency of ET suctioning in ventilated neonates ^8^. Due to the limitation of objective evidence to give recommendations in the pediatric population, developing clinical trials with the methodological rigor implied in the realization of this study, contribute to scientific evidence. Because of few exclusion criteria and the type of institution in which this trial was performed, its results are applicable to whichever PICU. In conclusion, the results of this trial suggest that the protocol of as-needed ET suctioning has more advantages in logistics, is the most physiological, does not increase pediatric morbi- mortality and could have additional economic benefits.
