# Supplementary material for: As-needed endotracheal suctioning protocol vs a routine endotracheal suctioning in Pediatric Intensive Care Unit: A randomized controlled trial
Source: Colomb Med (Cali). 2018 Jun 30;49(2):148–53. doi: 10.25100/cm.v49i2.2273 (PMC6084919; doi:10.25100/cm.v49i2.2273)
Supplement: Related texts

# Research article: Protocolo de aspiración endotraqueal según necesidad vs aspiración endotraqueal de rutina en una Unidad de Cuidado Intensivo Pediátrico: Un ensayo clínico controlado

## Introducción

El porcentaje de intubación y ventilación mecánica en la Unidad de Cuidado intensivo pediátrico (UCIP) varía entre 17 y 64% con una mediana de duración de 7 días ^1^. La presencia del tubo endotraqueal (TET) y la inadecuada humidificación del aire inspirado pueden causar irritación de la vía aérea con el consiguiente incremento en la producción de secreciones. La acción mucociliar inefectiva, por la sedación y el impedimento para cerrar la glotis por la presencia del TET, afectan el mecanismo de la tos causando retención de secreciones, obstrucción de la vía aérea, aumento de la resistencia de la vía aérea, disminución de la ventilación y del flujo de aire y finalmente el incremento en el trabajo respiratorio. Para prevenir lo anterior, es necesario remover las secreciones acumuladas en el TET, la tráquea y las vías respiratorias inferiores mediante la aspiración endotraqueal (AE) ^2^. Aunque la técnica es relativamente simple, varios estudios han descrito eventos adversos asociados a este procedimiento, entre los cuales se destacan la hipoxemia, con disminución en la saturación de oxígeno de hasta un 30%, presencia de arritmias, cianosis, cambios en la presión arterial, riesgo de introducir patógenos en el tracto respiratorio, extubación accidental y en algunos casos, aunque de manera infrecuente, la muerte ^3^. 

En la literatura existen recomendaciones relacionadas con la presión de succión, los sistemas de aspirado, la duración del procedimiento, la profundidad de inserción del catéter y el tamaño del mismo, pero existe controversia con respecto a la frecuencia para realizar la AE, así como el impacto de la misma en la duración del soporte ventilatorio, la duración de la estancia en la unidad de cuidados intensivos pediátricos (UCIP), la incidencia de infecciones asociadas al cuidado de la salud y las extubaciones accidentales ^2^. Las ultimas guías de la “American Association of Respiratory Care” (AARC), recomiendan fuerte, pero con baja calidad de la evidencia, la práctica de la AE sólo ante la presencia de secreciones ^4^. Esta recomendación se basó en tres ensayos clínicos, uno realizado en adultos y los otros dos en niños con muy bajo peso al nacer ^5^^-^^7^, todos implementados hace más de 12 años.

Teniendo en cuenta los múltiples riesgos que representa la AE para la población pediátrica, los cuales se aumentan con su frecuencia, el hecho de disminuir el número de aspiraciones se podría reflejar en una disminución de las extubaciones accidentales y sus consecuencias, así como los efectos negativos inherentes a la aspiración.

En este ensayo clínico se comparan ambos protocolos de AE, para identificar si hay diferencias en la frecuencia de la morbilidad asociada a la aspiración, los días de ventilación mecánica, la duración de la estadía en la UCIP, la incidencia de neumonía asociada al ventilador (NAV) y la mortalidad en la UCIP.

## Materiales y Métodos

### Diseño del estudio y participantes

Este ensayo clínico pragmático, paralelo, abierto, controlado y aleatorizado consideró para la asignación al azar a todos los niños mayores de un mes hasta los 14 años más 364 días, hospitalizados en la UCIP que necesitaron intubación endotraqueal y ventilación mecánica, cuyos padres aceptaron firmar el consentimiento informado. Se excluyeron del estudio aquellos pacientes con necesidad de ventilación mecánica de alta frecuencia, debido al riesgo de despresurización por la práctica de la aspiración y a la dificultad de visualizar el patrón de presencia de secreciones en niños sometidos a este tipo de ventilación.

Este estudio se desarrolló durante un período de 6 meses en el Hospital Universitario Pablo Tobón Uribe, ubicado en Medellín-Colombia, el cual cuenta con 313 camas, 10 en la UCIP, con 72 egresos al año. El protocolo del estudio fue revisado y aprobado por el Comité Institucional de ética.

### Asignación al azar 

La asignación al azar al grupo de AE a necesidad o rutinaria, se obtuvo mediante el uso del programa RAND mediante una secuencia de números aleatorios distribuidos por bloques permutados de tamaño 4, 6 y 8 generada por la investigadora principal que no hacía parte del equipo asistencial. El ocultamiento de la secuencia se realizó por medio de sobres opacos sellados, numerados secuencialmente en la Unidad de Investigación del Hospital. Los pacientes fueron asignados a los grupos inmediatemente después de la intubación 

### Intervenciones

Se tuvieron en cuenta las normas de la técnica aséptica para AE. Posterior a la obtención del consentimiento informado por los padres, la práctica de la AE fue realizada en ambos grupos utilizando un catéter Trach Care^™^ la cual era introducida en el TET evitando tocar la vía aérea y aplicando presión negativa, entre 20 y 40 mmHG, según la edad del paciente, por un período máximo de 10 segundos. Esta técnica fue repetida hasta que la vía aérea quedara permeable, lo cual fue verificado mediante la observación de ausencia de secreciones en el aspirador y del patrón del flujo. El tamaño del catéter a utilizar estaba protocolizado, como norma, se asignó un catéter con el doble del número del tubo que tuviera el niño: para los tubos número 3 se usó un catéter número 6, para los tubos número 4 se usó un catéter número 8 ^2^. 

En el grupo de Intervención, la práctica de la aspiración se realizó *a necesidad* según la presencia de uno o varios de los siguientes criterios visualizados en el monitor del ventilador o con la observación directa:

- Saturación de oxígeno menor de 90% (desaturación arterial) no secundaria a movimiento, o pérdida de la onda de pulso, de al menos un minuto de duración y con saturaciones previas normales.
- Volumen corriente menor o igual a 5 mL/kg de lo que el paciente movilizaba antes de la AE y cuya disminución no fuera secundaria a manipulación previa. Para cada paciente el pediatra de turno calculó el volumen corriente absoluto ideal en mL/kg y el volumen corriente mínimo permitido, el cual se programó en la alarma del ventilador. Se consideró la caída en el volumen corriente teniendo como referencia el volumen corriente absoluto ideal que tenía el paciente. Este valor se tomó de las notas de la evolución del terapeuta respiratorio y del pediatra. No se consideraron parámetros de presión como PEEP y control de presión.
- Curva flujo/ tiempo que mostrara la presencia de patrón en “dientes de sierra o melladura” que indicaran presencia de secreciones en el tubo. Se descartaba la presencia de agua en el tubo del ventilador.
- Asincronía del patrón respiratorio con el ventilador: patrón respiratorio irregular y descoordinado con el modo ventilatorio.
- Secreciones visibles y audibles.

En el grupo Control la práctica de la aspiración se realizó de *forma rutinaria* cada dos horas. En este grupo la frecuencia de la aspiración podía ser modificada si el paciente presentaba cualquiera de los criterios definidos para el grupo de intervención, permaneciendo en el brazo del tratamiento asignado.

Debido a que no fue posible el enmascaramiento de las intervenciones, se cuantificó la frecuencia de las siguientes co-intervenciones que se encuentran protocolizadas dentro de los cuidados básicos de enfermería y que se podrían modificar por el conocimiento de los grupos y que a la vez pueden afectar los resultados esperados: cambios de posición, baño en cama, fijación del TET y sedación. Esta evaluación la realizaron personas diferentes al equipo investigador. 

### Desenlaces

Se estableció un desenlace primario de morbilidad, el cual fue compuesto por cuatro componentes definidos así:

- Hipoxemia como la caída en la saturación del 10% del valor previo al procedimiento, presentada durante o inmediatamente terminaba la aspiración.
- Extubación accidental paro respiratorio.
- Arritmias como la alteración del ritmo cardíaco de base, presentadas durante o inmediatamente terminaba la aspiración y consideradas como: bradicardia si la frecuencia cardíaca era menor del percentil 5 para la edad o taquicardia si la frecuencia cardíaca era mayor del percentil 90 para la edad o había un incremento del 10% para aquellos pacientes que se encontraban taquicárdicos antes de la aspiración.
- Extrasístoles como la presencia de latidos adelantados respecto a la frecuencia normal, identificados por la auxiliar de enfermería y verificados por el pediatra y grabados en los monitores. Los datos para el registro de estos eventos adversos se tomó del monitor central CNS Nihon Kohden V 01-55 9701J con ayuda de la versión en español del software Vorax 5001.

Se definieron los siguientes desenlaces secundarios:

- Neumonía asociada al ventilador cuya definición se basó en los criterios del “*Center Disease Control*” (CDC) de Atlanta y el diagnóstico lo verificó el Comité de Prevención de infecciones.
- Días de ventilación mecánica y estancia en la UCIP contabilizados todos los días a las 7 horas y 30 minutos. Esta medición estuvo a cargo del Comité de prevención de infecciones de lunes a viernes. Durante fines de semana y festivos, la responsable de la medición era la enfermera jefe del servicio. Para el cálculo, se tuvo en cuenta la fecha, hora de ingreso y egreso a la UCIP en el registro de historia clínica electrónica.

Las mediciones de los parámetros cardiopulmonares fueron realizados por la auxiliar de enfermería, la profesional de enfermería, la terapeuta respiratoria o el pediatra y fueron consignados en una hoja de registro estandarizada. Un médico diferente al grupo tratante asignó los diagnósticos de acuerdo al análisis hecho a tales datos. Se consideraron variables adicionales como: edad en meses, sexo, peso en kilogramos, diagnóstico de ingreso a la UCIP consignado en la historia clínica electrónica por el pediatra y el PRISM (*Probability Risk Infant Score Mortality*) calculado durante las primeras 24 horas de ingreso a la UCIP. 

Los desenlaces incluidos fueron solo los que ocurrieron durante la estancia en UCIP.

### Tamaño de muestra 

Los cálculos se basaron en hallazgos de la literatura. Se asumió una proporción de eventos en el grupo de control de 45% y una disminución esperada de 25% en el grupo de intervención ^8^^,^^9^. El error alfa se estableció en un nivel de significanción de 0.05 y el error beta en 0.2. Teniendo en cuenta que se trataba de mediciones repetidas, se hizo un cálculo de tamaño de muestra con un coeficiente de correlación intracluster (CCI) de 0.1. Esperando tener un mínimo de 20 aspiraciones por cada niño incluido en el estudio, el número mínimo de pacientes requeridos por grupo para cumplir estos criterios fue de 45. El programa utilizado para el cálculo fue Sampsize versión 1.0.2.

### Análisis estadístico

La unidad de asignación al azar fueron los pacientes y la unidad de análisis fueron las aspiraciones. Para aquellas características basales de la población representadas en variables categóricas se calcularon frecuencias absolutas y relativas. Para las variables continuas, se evaluó la distribución normal de los datos por medio de las medidas de tendencia central, las medidas de distribución, los gráficos, y la prueba de Shapiro-Wilk. Debido a que la distribución de los datos no fue normal, se utilizaron medianas con su respectivo rango intercuartílico (RIC). Para el análisis bivariado de las variables cualitativas, se utilizó el estadístico de Chi^2^. En aquellos casos que los valores esperados en una celda fueron menores que 5, se realizó corrección por continuidad. Las variables cuantitativas se compararon con la prueba U de Mann Withney. Para ambos tipos de variables, un valor de p menor de 0.05 fue considerado como diferencia estadísticamente significativa. Para el cálculo del riesgo relativo del desenlace primario compuesto, se consideró que las respuestas estaban correlacionadas. Fue necesario modelar los datos utilizando una técnica para desenlaces dicotómicos con un modelo de ecuaciones estimables generalizadas (GEE). Dentro del modelo, el número que identificó cada paciente en la base de datos representó la variabilidad entre-sujeto y las mediciones de AE representó la variabilidad intra-sujeto. El tipo de modelo elegido fue el de respuesta logística binaria. La variable dependiente fue el desenlace primario compuesto, la categoría de referencia fue el grupo control y como factores predictores se ingresaron el método de AE (rutinario o a necesidad) y el número de AE. Se evaluaron las estructuras de las matrices de correlación de trabajo y el modelo elegido fue aquel que presentó los menores valores para el error típico y para el criterio de cuasi-verosimilitud de la bondad de ajuste^10^. Todos los análisis fueron por intención de tratamiento, todos los pacientes admitidos permanecieron en el estudio, independiente de la duración final de la intubación y se utilizó el programa “Statistical Package for the Social Sciences” (SPSS) versión 19.0 para Windows. 

## Resultados

Durante el periodo de estudio, se admitieron 183 pacientes a la UCIP. De estos pacientes, 92 fueron asignados al azar para recibir AE a necesidad o rutinaria. Finalmente 45 pacientes recibieron AE a necesidad y 45 recibieron AE rutinaria. No hubo pérdidas de seguimiento debido a que cada paciente se siguió hasta el alta de la UCIP (Fig. 1).

**Figura 1:** Flujo de pacientes

De la población reclutada, 52% (48/92) fueron hombres. La mediana de edad fue 40.5 meses (RIC: 8.3-107.1). Los motivos de ingreso a la UCIP fueron las enfermedades infecciosas en 16% (15), enfermedades congénitas en 12% (11), enfermedades neurológicas y quirúrgicas en 11 % (10), enfermedades cardiovasculares en 9% (8), enfermedades respiratorias en 8% (7) y 34% (31) ingresaron por otras causas. De los participantes admitidos, 23% (21) falleció. La mediana del puntaje PRISM para la población asignada al azar fue de 22 puntos (RIC: 15-26). La mayoría de los niños (44%) al momento del ingreso presentó riesgo bajo de fallecer (PRISM <20 puntos). La mediana de la duración de la ventilación y de la estancia en UCIP, fue de 2.5 días (1.0-5.7) y 5.8 días (2.0-14.6), respectivamente. El 98% de los participantes recibió sedación, baño en cama y cambios de posición de acuerdo con el protocolo de cuidados de enfermería. El protocolo de fijación de TET, se aplicó al 96% (88) de los participantes, pues hubo cuatro participantes que ingresaron con traqueostomía. 

Las características clínicas y demográficas de los pacientes fueron similares entre los dos grupos (Tabla 1). La mediana de AE por paciente para el grupo a necesidad y de control fue de 8 AE (RIC: 4.0-13.0 vs 4.0-14.0). La media del tiempo entre cada aspiración en el grupo de AE a necesidad fue 3.8 horas. El desenlace primario compuesto estuvo presente en 22 (47%) niños del grupo de intervención y 25 niños (55%) del grupo control (RR= 0.84; IC 95%: 0.56-1.25). Al final del seguimiento, se presentó el desenlace primario compuesto en 35 (5.8%) de las 606 AE realizadas al grupo de intervención y en 48 (7.4%) de las 649 AE realizadas al grupo control (Tabla 2). Después de evaluar las diferentes estructuras de matriz de correlaciones, se eligió la estructura independiente y se obtuvo un OR para el desenlace primario compuesto de 0.8 con un intervalo de confianza del 95% de 0.57-1.3 (*p*= 0.31). Sólo se presentó un caso de neumonía asociada al ventilador en el grupo control sin diferencias estadísticamente significativas entre los grupos (*p*= 0.98). En el grupo de intervención la mediana de la duración de la ventilación mecánica fue de 2.5 días (1.0-5.0) versus 2.4 días (0.8-6.4) en el grupo control sin diferencias estadísticamente significativas (*p*= 0.93). La duración de la estancia en la UCIP fue mayor para el grupo de intervención con una mediana de 6.0 días (1.8-12.6) versus 4.4 días (2.4-22) en el grupo control (*p*= 0.76). De forma exploratoria se encontró que el 22.8% (21) del total de participantes falleció. La mayoría de estos (27%), se encontraba en el grupo control (*p*= 0.39). No hubo seguimiento después del egreso de la UCIP.

**Tabla 1:** Características clínicas y demográficas de los participantes

Características	Protocolo AE a necesidad (intervención) n= 47	Protocolo AE de rutina (control) n= 45
Hombres. n (%)	26 (55.3)	22 (48.9)
Edad en meses. Mediana (P25-75)	34 (7-104)	56 (14-108)
Peso (kg). Mediana (P25-75)	15 (7-31)	15 (10-30)
PRISM. Mediana (P25-75)	23 (14-28)	23 (17-27)
Diagnóstico de ingreso n(%)		
Enfermedad respiratoria	5 (10.6)	2 (4.4)
Enfermedad neurológica	7 (14.9)	3 (6.7)
Post-procedimiento quirúrgico	7 (14.9)	3 (6.7)
Malformaciones congénitas	7 (14.9)	4 (8.9)
Enfermedad Cardiovascular	3 (6.4)	5 (11.1)
Infección	4 (8.5)	11 (24.4)
Otras	14 (29.8)	17 (37.8)

**Tabla 2:** Resultados

Desenlace	Protocolo AE a necesidad (n=606)^**§**^	Protocolo AE de rutina (n=649)	OR(IC95%)^‡^
Desenlace compuesto primario †	35 (5.8%)	48 (7.4%)	0.8 (0.5-1.3)
Arritmias	26 (4.4%)	41 (6.4%)	0.7 (0.4-1.2)
Hipoxemia	10 (1.7%)	7 (1.1%)	1.5 (0.5-4.4)
Extubation accidental	0	0	
Paro cardio respiratorio	0	0	

§ Número de aspiraciones

‡ OR calculado teniendo en cuenta la correlación entre las mediciones

†Hipoxemia, arritmia, extubación accidental y paro cardiorespiratorio

## Discusión

Este ensayo sugiere que la aplicación de un protocolo de AE a necesidad comparado con uno de AE rutinaria, no presenta diferencias en la frecuencia de la morbilidad para los niños que ingresan a la UCIP. Aunque se reconoce que para declarar la igualdad entre dos intervenciones es necesario realizar un ensayo de no inferioridad, la pequeña diferencia encontrada entre los dos grupos, con mínima significación clínica, sugiere que ambas intervenciones son similares respecto a los eventos adversos, consistente con las recomendaciones actuales de las pautas clínicas.

Las guías de la “American Association Respiratory Care” (AARC) para la práctica de la AE en pacientes con ventilación mecánica y vía aérea artificial recomiendan que se debe evitar la AE de rutinaria, apoyándose en los resultados de tres ensayos clínicos, sin embargo uno fue realizado en adultos ^7^ y dos en neonatos de de muy bajo peso al nacer con síndrome de dificultad respiratoria ^5^^,^^6^. La frecuencia de AE así como los desenlaces evaluados en esos estudios fueron diferentes a los evaluados en nuestro estudio. Una revisión de 5 estudios viejos, con solo un ensayo clínico, que evaluó la frecuencia de la aspiración en neonatos y niños concluyó que la información para soportar el uso de aspiración a necesidad en ellos es escasa ^11^. Los resultados de nuestra investigación aportan información adicional para la población pediátrica, para la cual la frecuencia de la aspiración no se ha evaluado previamente en ensayos clínicos.

Existen varios estudios observacionales en la población pediátrica, que han descrito la incidencia de eventos adversos asociados a la práctica de la AE, los cuales fueron revisados recientemente ^12^. Algunos autores han descrito que la hipoxemia es el evento adverso más frecuente durante la práctica de la AE (20 %) ^3^^,^^13^, este hallazgo difiere de lo encontrado en nuestro estudio, donde las arritmias, principalmente la bradicardia, fue la complicación más frecuente como ha sido reportada en otros estudios ^14^^,^^15^.

La ausencia de extubación accidental durante la práctica de la AE en este estudio difiere de lo reportado en los trabajos, donde la práctica de procedimientos como la AE fue descrita como uno de los factores de riesgo para la extubación accidental y se ha descrito que la manipulación para la AE al momento de la extubación no planeada en la UCI neonatal fluctúa entre el 17% y 30% de todos los pacientes que tuvieron extubaciones no planeadas ^16^. 

Algunos autores han descrito incremento en los días de ventilación mecánica y estancia en la UCIP como consecuencia de la extubación accidental ^17^. Nuestro estudio no demostró diferencias en ambas duraciones, uno de los aspectos que pudo influenciar este resultado podría ser la ausencia de extubaciones accidentales. Las diferencias en la frecuencia de los desenlaces secundarios de nuestro estudio con los estudios observacionales, posiblemente se explican por aspectos metodológicos.

No hubo esfuerzos adicionales ni necesidad de que más personal para practicar el protocolo a necesidad. Utilizando este protocolo, el tiempo entre las aspiraciones se duplicó. El tiempo liberado fue utilizando para proporcionar otros cuidados que den más beneficios para los pacientes. Sin embargo, este resultado no se midió objetivamente teniendo en cuenta que no era el objetivo del estudio.

Una de las limitaciones para este trabajo fue la baja frecuencia del desenlace primario, menor que el estimado de esos reportes de la literatura, lo cual afectó el poder del estudio, sin embargo, la diferencia encontrada entre los dos grupos es muy pequeña y también fue menor que la estimada de los estudios observacionales, por lo que se considera plausible aceptar que la falta de detección de diferencias significativas en la frecuencia de desenlaces entre los dos los grupos no se debió a la disminución del poder del estudio. Una segunda limitación fue la falta de cegamiento, que es imposible evitar considerando el tipo de intervención implementada, pero se trató de controlar evaluando el resultado por el personal independiente del equipo de investigación e indirectamente evaluando la posibilidad de que este conocimiento induciría otra las co-intervenciones diferenciales, que no aparecieron. También hubo dificultad para definir de manera objetiva los componentes del desenlace principal.

Una revisión Cochrane reciente de la frecuencia de aspiración endotraqueal para la prevención de la morbilidad respiratoria en recién nacidos ventilados, encuentró que la frecuencia ideal de la AE se ha investigado en pocos estudios, identificó sólo un estudio aleatorio controlado de 1991 para incluir en la revisión y concluye que no hubo suficiente evidencia para identificar la frecuencia ideal de la AE en neonatos ventilados.^8^ Debido a que la evidencia objetiva para dar recomendaciones en la población pediátrica es limitada, la realización de ensayos clínicos con el rigor metodológico empleado en la realización de este estudio permite aportar a la evidencia científica. Por los pocos criterios de exclusión y el tipo de institución en la que fue realizado este trabajo, sus resultados son aplicables a cualquier UCIP. En conclusión, los resultados de este trabajo sugieren que el protocolo de AE a necesidad tiene más ventajas en aspectos logísticos, es el más fisiológico, no incrementa la morbimortalidad y podría tener beneficios económicos adicionales.
